# Pathways of Lipid Metabolism in Marine Algae, Co-Expression Network, Bottlenecks and Candidate Genes for Enhanced Production of EPA and DHA in Species of Chromista

**DOI:** 10.3390/md11114662

**Published:** 2013-11-22

**Authors:** Alice Mühlroth, Keshuai Li, Gunvor Røkke, Per Winge, Yngvar Olsen, Martin F. Hohmann-Marriott, Olav Vadstein, Atle M. Bones

**Affiliations:** 1 Department of Biology, Norwegian University of Science and Technology, Trondheim 7491, Norway; E-Mails: alice.muehlroth@ntnu.no (A.M.); keshuai.li@ntnu.no (K.L.); per.winge@ntnu.no (P.W.); yngvar.olsen@ntnu.no (Y.O.); 2 Department of Biotechnology, Norwegian University of Science and Technology, Trondheim 7491, Norway; E-Mails: gunvor.røkke@ntnu.no (G.R.); martin.hohmann-marriott@ntnu.no (M.F.H.-M.); olav.vadstein@ntu.no (O.V.)

**Keywords:** *Phaeodactylum tricornutum*, long chain polyunsaturated fatty acid synthesis, metabolic engineering, elongases, desaturases, acyl-CoA synthetases, acyltransferases

## Abstract

The importance of *n*-3 long chain polyunsaturated fatty acids (LC-PUFAs) for human health has received more focus the last decades, and the global consumption of *n*-3 LC-PUFA has increased. Seafood, the natural *n*-3 LC-PUFA source, is harvested beyond a sustainable capacity, and it is therefore imperative to develop alternative *n*-3 LC-PUFA sources for both eicosapentaenoic acid (EPA, 20:5*n*-3) and docosahexaenoic acid (DHA, 22:6*n*-3). Genera of algae such as *Nannochloropsis*, *Schizochytrium*, *Isochrysis* and *Phaedactylum* within the kingdom Chromista have received attention due to their ability to produce *n*-3 LC-PUFAs. Knowledge of LC-PUFA synthesis and its regulation in algae at the molecular level is fragmentary and represents a bottleneck for attempts to enhance the *n*-3 LC-PUFA levels for industrial production. In the present review, *Phaeodactylum tricornutum* has been used to exemplify the synthesis and compartmentalization of *n*-3 LC-PUFAs. Based on recent transcriptome data a co-expression network of 106 genes involved in lipid metabolism has been created. Together with recent molecular biological and metabolic studies, a model pathway for *n*-3 LC-PUFA synthesis in *P. tricornutum* has been proposed, and is compared to industrialized species of Chromista. Limitations of the *n*-3 LC-PUFA synthesis by enzymes such as thioesterases, elongases, acyl-CoA synthetases and acyltransferases are discussed and metabolic bottlenecks are hypothesized such as the supply of the acetyl-CoA and NADPH. A future industrialization will depend on optimization of chemical compositions and increased biomass production, which can be achieved by exploitation of the physiological potential, by selective breeding and by genetic engineering.

## 1. Introduction

Long chain polyunsaturated *n*-3 fatty acids (LC-PUFAs) are of increasing interest, due to their many positive effects for human health and their use as feed for fish farming. Until now, seafood has been the main source of *n*-3 LC-PUFAs; however, as seafood harvesting is at peak production, alternative sustainable sources for *n*-3 LC-PUFAs should be developed. Most fishes cannot produce *n*-3 LC-PUFAs themselves, but are channelled up the marine food chain, with microalgae as the primary producers [1]. Therefore, it is not surprising that marine microalgae have been targeted as potential candidates for industrial production of *n*-3 LC-PUFAs such as eicosapentaenoic acid (EPA, 20:5*n*-3) and docosahexaenoic acid (DHA, 22:6*n*-3) [2,3,4,5].

Many of the *n*-3 LC-PUFA-producing algae belong to the Chromista kingdom, a diverse group of microorganisms that includes divisions like cryptomonads, haptophytes and heterokonts [6]. The classification is based on the hypothesis that chloroplasts of all Chromista arose from a single secondary endosymbiotic event between a eukaryote and a red alga-like organism [6,7]. Interestingly, the heterotrophic thraustochytrid, also belonging to Chromista, have lost their photosynthetic capacity but have retained a vestigial chloroplast and their ability to synthesize and store *n*-3 LC-PUFAs, as chloroplasts are the site of lipid synthesis in alga.

At present, mostly thraustochytrids like *Schizochytrium* spp. are used in industrial-scale *n*-3 LC-PUFA production as they can reach a DHA content of 43% (cell dry weight) and have a productivity of up to 7.2 g of DHA per liter of culture and per day [8,9]. The close relationship between the thraustochytrids and photosynthetic microalgae of the Chromista kingdom suggests a high potential for these microalgae as an alternative *n*-3 LC-PUFA source in the future. Additionally, algae have advantages such as consuming carbon dioxide, growing in salt water on marginal land and thereof no compete with the agriculture industry or freshwater use [10]. The major genera of commercial microalgae are to date Chromista [11,12,13]. EPA-producing heterokonts include the photoautotrophic commercial-used *Nannochloropsis* spp. [14], *Monodus subterraneus* [15], *Nitzschia* spp. [16] and the model diatom *Phaeodactylum tricornutum* [17]. Whereas DHA-producers include *Isochrysis galbana* [11,18] and the thraustochytrids *Aurantochytrium* spp. [19], *Thraustochytrium* spp. [11,12], and *Schizochytrium* spp. [20]. The amount of *n*-3 LC-PUFAs produced by these organisms depends on the environmental conditions. Exposing the algae to environmental stresses such as nitrate starvation [17], increased salinity [20], changes in light intensity [15] or variations in the amount and composition of carbon [14,20] can increase both lipid synthesis and accumulation and also the composition of *n*-3 LC-PUFAs themselves. Several recent reviews have dealt with this topic for different algae [9,21,22,23,24,25]. For instance, the eustigmatophyceae *Nannochloropsis* has under mild growth conditions an EPA content of 1.6% of cell dry weight, while subjecting *Nannochloropsis oceanica* to high light and N-limitation an EPA content of 2.6% (cell dry weight) can be reached [14,18,26]. However, the low production of *n*-3 LC-PUFAs per culture volume and day, mainly due to low biomass density, makes photosynthetic production of *n*-3 LC-PUFA not profitable at present [13].

To improve the *n*-3 LC-PUFAs or the lipid bodies, triacylglycerides (TAGs), productivity in algae two main strategies exist; (1) Increase the content of desired lipids per unit of biomass and (2) Increase the biomass density of the given strain to maximize biomass per culture volume or area. Optimization of growth conditions that increase *n*-3 LC-PUFAs is challenging, as LC-PUFAs and TAGs accumulate under abiotic stress, which in turn decreases the biomass yield. Besides optimizing physical growth conditions approaches such as selection/breeding and metabolic engineering can be used to enhance the lipid yield. These high level skill approaches have advantages and disadvantages, while genetic engineering requires significant resources in establishing a suitable transformation of functional plasmids, selection processes require a sustainable breeding and selecting program [21,22]. A combination of approaches like metabolic engineering and selective breeding have been shown to be successful in plants by genetic engineering of *n*-3 LC-PUFAs and classical mutation strategies to bypass bottlenecks [23,24,25]. Successful approaches must be based on identification of genetic drivers influencing both qualitative and quantitative aspects of the lipid metabolism in Chromista. Even though genetic drivers are applicable for different approaches, we will in this review focus on the metabolic engineering approach because it has been discussed by Khozin-Goldberg*et al.* (2011) as a strategy to obtain a high yield of *n*-3 LC-PUFA by microalgae and reviews focused on engineering of lipid metabolism in algae with emphasis on TAG accumulation [22,27,28,29,30].

In this review, we address the synthesis and compartmentalization of *n*-3 LC-PUFAs in Chromista by mapping the lipid metabolism for the model diatom *P. tricornutum*. Genomic data have been analyzed and a co-expression network has been assembled. Several pathways have been predicted and candidate genes and bottlenecks were identified in order to find strategies for improve the *n*-3 LC-PUFAs in commercially used Chromista such as *Nannochloropis* or *Isochrysis*. In doing so, we have identified and compared parts of the known EPA and DHA pathways of biotechnological important Chromista species which differ from the lipid pathway of *P. tricornutum*.

## 2. Long Chain Polyunsaturated Fatty Acids (LC-PUFAs) for Human Health

Linoleic acid (LA, 18:2*n*-6) and α-linolenic acid (ALA, 18:3*n*-3) are essential fatty acids (FAs) for vertebrates [31,32] and are precursors for the LC-PUFA of the *n*-6 group arachidonic acid (ARA, 20:4*n*-6) and of the *n*-3 group EPA and DHA. Humans have the capability to synthesize EPA (20:5*n*-3) and DHA (22:6*n*-3) from ALA (18:3*n*-3), but the capacity is too low to provide sufficient amounts of these *n*-3 LC-PUFAs for maintenance of mental and cardiovascular health [33,34]. Some fresh water and diadromous fish species may survive on diets containing only C18 PUFA, whereas marine fish have a strict requirement for long chain PUFAs that e.g. play important roles in osmoregulation for animals in different aquatic environments [32]. ARA (20:4*n*-6), EPA (20:5*n*-3) and DHA (22:6*n*-3) are also required for normal growth, development of specific and nonspecific immunity, and stress tolerance of marine fish, especially at early stages [35,36,37].

The membranes of the brain contain high amounts of ARA (20:4*n*-6) and DHA (22:6*n*-3) in the phospholipids, and are important for optimal visual and cognitive development and functioning. Fish larvae and human infants have high requirement of LC-PUFA, probably due to an immature digestive/absorptive system and the need for fast growth and development, especially for neural and visual tissues [38]. The beneficial health effects of *n*-3 FAs, particularly *n*-3 LC-PUFAs, have been extensively studied [13,39,40,41]. Positive effects include anti-viral, anti-bacterial and anti-fungal effects [42,43]. These benefits appear to be related to the alternations of fluidity in membrane phospholipids composition and function, gene expression and eicosanoid production [44]. In general, it is recommended to increase the dietary *n*-3 FAs intake for the human population, but the recommendations vary in different countries because of different dietary background and cultural traditions [45,46]. For instance, the American Dietetic Association recommends 500 mg/day of EPA + DHA [47], whereas the Norwegian authorities propose 250 mg/day of EPA + DHA for older children and adults and 0.5 E% (percent of total energy intake) of total *n*-3 FAs, 0.10 g/day of DHA (22:6*n*-3) for infants and small children (6–24 months) [48]. Beside the absolute amount of PUFAs that is required, the ratio of *n*-6/*n*-3 FAs is considered to be very important for development and health of both humans and fish. EPA (20:5*n*-3) and ARA (20:4*n*-6) are precursors for the synthesis of hormones named eicosanoids which are involved in many cell regulatory functions. EPA-derived eicosanoids have potent anti-angiogenic effects, whereas ARA-derived metabolites have pro-angiogenic effects. Because EPA (20:5*n*-3) and ARA (20:4*n*-6) compete for the common enzymes, cyclooxygenases, lipooxygenases, and cytochrome P450, the ratio between *n*-6 and *n*-3 FAs seems to determine the ratio of the respective enzymatic products, EPA (20:5*n*-3) and ARA (20:4*n*-6) derived eicosanoids [44,49,50]. The ratio of *n*-6/*n*-3 FAs in typical Western diets is 15/1–16.7/1, whereas it is suggested that humans evolved on a diet with a ratio close to 1 [40]. A ratio of *n*-6/*n*-3 of 4:1 or less seems to reduce the risk of many chronic diseases, such as cardiovascular disease, colorectal cancer, breast cancer and asthma [40].

An overview of the content of *n*-6 and *n*-3 PUFAs in different organisms and food sources reveals that the *n*-6/*n*-3 ratios of commonly farmed and wild fish species in Norway and frequently used marine microalgae is in general very low (<1:2), whereas the ratio in red meat, chicken and plant oils is high (>4:1; Figure 1). A high percentage of *n*-3 FAs, e.g., >20%, is only found in marine organisms, and none of the marine organisms contain >15% *n*-6 FAs.

**Figure 1:**
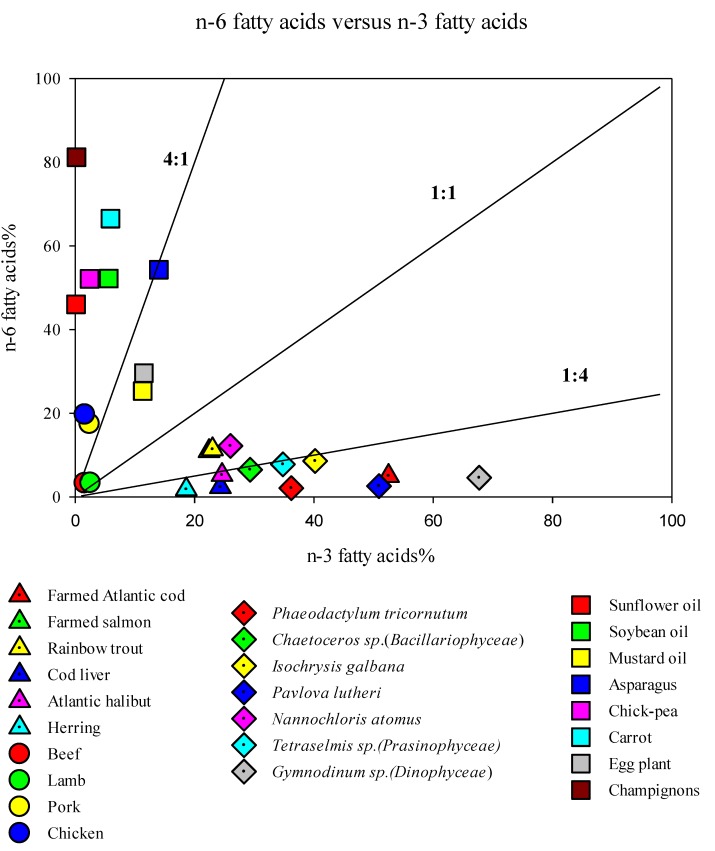
*n*-6 fatty acids (%) *versus* *n*-3 fatty acids (%) in different food sources and microalgae [51,52,53,54,55,56].

## 3. LC-PUFA Sources and the Need for Alternatives

The main source of *n*-3 LC-PUFAs for human consumption is currently marine fish and in particular the fish oils of fat fish. The world capture fisheries have remained stable since 1990, whereas aquaculture production has increased strongly [57]. The global production of fish oil decreased from 1.6 million tons in the late 1980s to 1 million tons in 2012, of which about 7% was used for human consumption and about 88% was used for aquaculture [48]. During the last decades, the aquaculture industry has started to replace fish oil and fish meal with plant seed meals and vegetable oils in order to secure growth in the production [58,59]. Studies suggest that salmonids and gilthead sea bream can be grown with 100% of blended vegetable oils, without any serious effect on growth rates [60,61]. However, the EPA (20:5*n*-3) and DHA (22:6*n*-3) levels in the flesh of the fish fed were reduced by feeding high levels of vegetable oils, and the beneficial effects of fish consumption to the human consumer is therefore reduced [32]. Meanwhile, the positive health effects of the *n*-3 PUFAs, especially EPA (20:5*n*-3) and DHA (22:6*n*-3) have become widely accepted, and the human consumption for *n*-3 PUFAs in various products is increasing. Fortified foods (milk, yogurt, eggs and breads) and dietary supplements (cod liver oil, fish oil capsules) with oils that are rich in EPA (20:5*n*-3) and DHA (22:6*n*-3) have become widely used [62]. As traditional fisheries cannot increase in size, there is a search for alternative *n*-3 LC PUFA sources for the future expansion of both marine aquaculture and human consumptions. Proposed alternatives are large stocks of herbivore copepods, krill in the world’s oceans and microalgae [34,58].

## 4. Chromista and Their LC-PUFA Content and Function

Chromista include promising candidate species for commercial production of *n*-3 LC-PUFA but a deeper understanding of the synthesis, composition and function of fatty acids (FAs) in algae is needed for a knowledge-based future industrial production. In general, saturated and monounsaturated FAs are predominant in the FA profile of Chromista, and with a minor amount of ARA (20:4*n*-6) and a high level of one *n*-3 LC-PUFA, EPA (20:5*n*-3) and DHA (22:6*n*-3). The total FA composition is highly variable from species to species. In this section an overview of LC-PUFA occurrence in Chromista cells and the function of LC-PUFAs will be given.

LC-PUFAs in Chromista are mainly associated with the membranes or found in the storage compartments as TAGs. Membrane systems in algae can be divided into the photosynthetic active thylakoid membrane and the structural membranes surrounding compartments for example the chloroplast or the endoplasmic reticulum (ER). In membrane lipids, PUFAs are esterified to glycerol and further processed to produce polar lipids.

**Figure 2:**
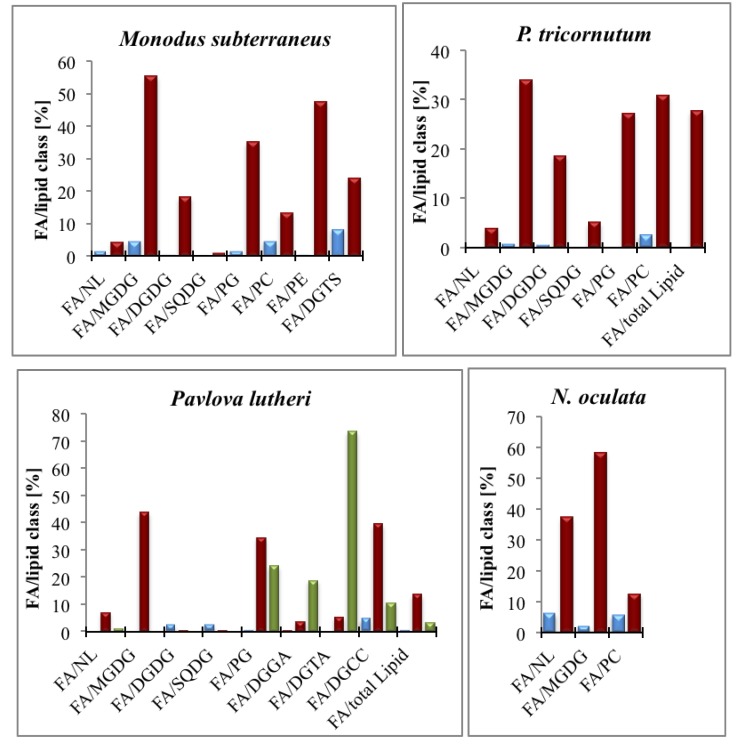
Content of LC-PUFAs of different lipid classes in selected Chromistans under different environmental conditions. Due to the experimental setups of the referred papers, not all lipid classes of the organisms could be assembled. *Monodus subterraneus* [67], *N. oculata* [68], *Pavlova lutheri* [69], *P. tricornutum* [70]. Blue: ARA (20:4*n*-6); red: EPA (20:5*n*-3); green: DHA (22:6*n*-3). MGDG: monogalactosyldiacylglycerol; NL: neutral lipids; DGCC: diacylglycerylcarboxyhydroxymethylcholine; DGDG: digalactosyldiacylglycerol; DGGA: diacylglyceryl glucuronide; DGTS: diacylglyceryltrimethylhomoserine; DGTA: diacylglycerylhydroxymethyl-trimethylalanine; SQDG: sulfoquinovosyldiacylglycerol; PC: phosphatidylcholine; PE: phosphatidylethanolamine; PG: phosphatidylglycerol.

In both the thylakoid and the non-photosynthetic membrane, the betaine-type glycerolipids (e.g., diacylglyceryltrimethylhomoserine (DGTS), diacylglycerylhydroxy-methyltrimethylalanine (DGTA) and diacylglycerylcarboxyhydroxymethylcholine (DGCC)) and the phosphoglycerides (e.g., phosphatidylcholine (PC) and phosphatidylethanolamine (PE)) are present [63]. The special component of the photosynthetic membrane is phosphatidylglycerol (PG) [27]. In all eukaryotic photosynthetic organisms, PGs contain an uncommon ∆^3^-trans hexadecenoic acid (16:1(3-t)) at their *sn*-2 position. PG-16:1(3-t) plays an important role in the LHCII trimerisation process and in the function of the photosystem [64]. The major components of the photosynthetic membrane are the glycosylglycerides, which are mainly uncharged polar lipids and far more abundant than phosphoglycerides. The glycosylglycerides vary in abundance from species to species [65]. Apparently diatoms possess negatively charged galactosylglycerides because of their higher levels of anionic sulphoquinovosyldiacylglycerol (SQDG) than land plants [66]. Monogalactosyldiacylglycerol (MGDG) and digalactosyldiacylglycerol (DGDG) are neutral lipids and contain either one or two galactose molecules linked to the *sn*-3 position of the 1,2-diacyl-*sn*-glycerol moiety [27]. Thus, FAs occupy the *sn*-1 and *sn*-2 positions, and the FA composition at these positions can be highly variable. Examples of PUFA distribution in different Chromista such as *N. oculata*, *P. tricornutum*, *Pavlova lutheri* and *M. subterraneus* under different environmental conditions are given in Figure 2. Figure 2 shows obvious differences in FA composition, for instance, *Pavlova lutheri* sustain high amount of DHA (22:6*n*-3), whereas *P. tricornutum* mostly contains EPA (20:5*n*-3).

Some diatoms, for instance, *P. tricornutum* and *Thalassiosira weissflogii*, contain *n*-3 LC-PUFA primarily in C20/C16 and C18/C16 forms of MGDG and DGDG; whereas the pennate diatom *Navicula perminuta* produce only C18/C16 and C18/C18 forms [71]. In plants, the FA combination of the galactosylglycerides can be traced back to their biosynthetic pathways; the so-called eukaryotic molecular species (C18/C18) of galactosylglycerides are synthesized outside the chloroplast in the eukaryotic pathway and the prokaryotic molecular species (C18/C16) are synthesized plastidial via the prokaryotic pathway [72]. Whereas in plants, clear separation of the galactosylglyceride origin is given, in algae most C20 FAs are synthesized outside of the chloroplast and are present in both molecular species of galactosylglycerides: the eukaryotic-like (C20/C20, C18/C18) and the prokaryotic-like (C18/C16, C20/C16) species [67,73]. Therefore, in algae it is suggested to refer to species of galactosylglycerides as “C20/C20” or “C20/MLC” (MLC = medium to long chain) rather than to eukaryotic- and prokaryotic-like molecular species [67]. In Chromista, the biological function of different combinations of FAs in galactosylglycerides remains unclear, but it will certainly affect the overall PUFA distribution and composition of the cell. However, some observations in other algae may be relevant. For instance in the red algae *Porphyridium curentum* a shift to lower temperatures decreases C20/C20 MGDG and increases C20/C16 MGDG [74]. This observation may be interpreted as a response to adjust the membrane fluidity of photosynthetic membranes. PUFAs in membranes are also important for adjusting membrane fluidity during shifts in salinity and light intensity [75]. It has been shown that a temperature-shift from 25 to 10 °C will enhance the proportion of PUFAs, especially EPA, by 120% in *P. tricornutum* [76].

TAGs are another but less likely *n*-3 LC-PUFA source in oleaginous algae. Most algae accumulate saturated and monounsaturated FAs in the TAGs under certain stress conditions such as P- and N-limitation and cell division arrest [77,78]. For example, in the eustigmatophyceae *N. oceanica* TAG accumulation increases while the EPA amount declines by 30% during N-deprivation [79]. Contrary to this, an increase in TAGs and incorporation of EPA in TAGs was observed in the diatom *T. pseudomonas* in the stationary growth phase [77]. TAGs are collected in lipid bodies in the cytoplasma and belong to the neutral or non-polar lipids together with, e.g., waxes, free FAs, and sterols [29]. Sukenik *et al.* (1991) proposed that TAG accumulation occurs in non-stress conditions during the day when cell division is at rest in order maximize harvest of photons and CO_2_ fixation [4]. The energy stored in TAG can be used to supply energy and reduced carbon in the dark phase. TAGs serve as a sink for excess electrons and bind and accumulate reduced carbon. Imbalance of the cellular C:N ratio, which occurs for example under N-limitation, leads to a rearrangement of the molecular pools. Whether carbohydrates or lipids are used as carbon/energy storage is dependent on the algae strain, the photosynthetic activity and environmental conditions [80,81]. In some algae, the major carbohydrate storage is the glucose polymer chrysolaminarin, which is used in heterokons such as the diatoms *Nitzschia sigmoida* and*Melosira varians* [82,83]. When cell division is not possible, the conversion of light energy into reduced carbon components, *i.e.*, fatty acids provides a useful mechanism to convert energy harvested by the photosynthetic machinery. The reduction of CO_2_ to the redox state of carbohydrates requires 2 mol of NADPH and ~3 mol of ATP, whereas the reduction of CO_2_ to the redox state of saturated fatty acids requires ~3 mol NADPH and ~4 mol ATP [84]. In addition to carbon and energy storage, the FA moieties of the lipid bodies are required for both the synthesis and the remodeling of PUFA rich thylakoid membranes [29].

PUFAs in algae possess several specific functions. One known function is to scavenge reactive oxygen, which interacts with the double bonds of the PUFA. This process, called lipid peroxidation, may play a role as an intermediate in cell signaling pathways [85]. Polar lipids such as inositol lipids or sphingolipids are also involved in such pathways [65]. Because LC-PUFAs are enriched in galactosylglycerides such as SQDG and M/DGDG in the thylakoid membranes, it is assumed that they contribute to the photosynthetic function of algae. For instance, studies of the green algae *Chlamydomondas reinhardtii* showed the importance of the glycosylglyceride SQDG for maintaining photosystem II functionality during environmental changes (e.g., temperature), and thus indicates, as well, a role of PUFAs in maintaining the photosynthetic machinery [75].

Advances in utilizing environmental factors to increase *n*-3 LC-PUFA in Chromista have been made, but in many cases the mechanisms behind this physiological plasticity, including regulation at the transcriptional level, are not understood. Information gained on the molecular level, could allow adaptation of algae, by selective breeding, metabolic engineering and physiological studies, as a *n*-3 producer on an industrial scale.

## 5. Eicosapentaenoic Acid (EPA, 20:5*n*-3) Synthesis in *Phaedoactylum tricornutum*

There are many studies of lipid accumulation in algae but there is still limited knowledge on the molecular mechanisms and the transcriptional regulations of the lipid metabolism. To exemplify the *n*-3 LC-PUFA synthesis and to find bottlenecks of the pathway, the model organism *P. tricornutum* has been used to assemble recent knowledge and to point out genetic drivers. For a better evaluation of the involvement of TCA cycle intermediates in the FA synthesis, studies of different Chromista have been included in the following section.

**Figure 3:**
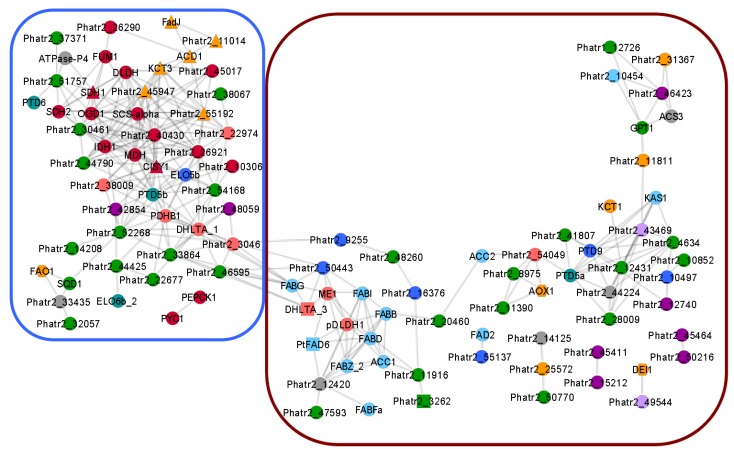
Co-expression network of 106 genes associated to the FA metabolism in *P. tricornutum*. The co-expression network can visually be divided into two subclusters. Subcluster 1 (blue, left square) contains mainly genes of the mitochondrial TCA cycle and β-oxidation. Subcluster 2 (red, right square) includes genes of the plastidial-located *de novo* FA synthesis and the endoplasmatic *n*-3 LC-PUFA biosynthesis. Color code: TAG biosynthesis (light purple); TCA cycle (red); ACCase (acetyl-CoA carboxylase); *de novo* FA and HTA (16:3*n*-4) synthesis (light blue); Predicted elongases and desaturases (dark blue); Predicted EPA pathway (turquoise); Acetyl-CoA precursors and transporter (light red); Acyl-CoA synthetases, ATpase4 (gray); Mitochondrial or peroxisomal located β-oxidation and FA elongation (yellow); Kennedy pathway, phospholipid-, glycerolipids, sphingolipid and sterol biosynthesis (green); Ca^2+^-dependent lipid-binding protein, amid hydrolase, DHHC palmitoyltransferase, serine incorporator, ATP-binding protein (ABC) transporter (purple). Shapes in the cluster indicate the localization of enzymes encoded by the gene: Triangle, mitochondria; Square, chloroplast; Diamond, peroxisome; Circle, no prediction. Transcription data of five microarray datasets from *P. tricornutum* submitted to GEO, NCBI (GSE12015, GSE17237, GSE31131, GSE42039 and GSE42514; [93,94,95,96,97]) were used to construct based on log2 expression ratios from the experiments, an unweighted co-expression network by using Cytoscape (version 2.8.3) and the force directed drawing algorithm [98]. The network represents 106 genes related to lipid metabolism with similar transcriptional profiles and includes 311 calculated gene-pairs with Pearson correlation values *r* > 0.85.

**Figure 4:**
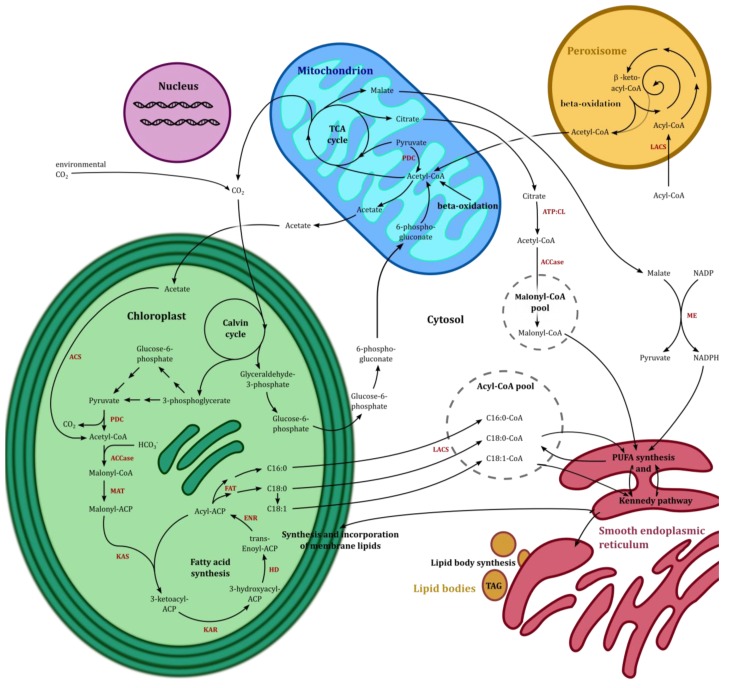
Simplified overview of the compartments, the main pathways and the metabolites in most Chromista; calvin cycle, fatty acid synthesis, tricarboxylic acid cycle, polyunsaturated FA pathway, β-oxidation and lipid synthesis shown in black arrows. Involved enzymes are shown in red: ACCase, acetyl-CoA carboxylase; ACS, Acyl-CoA synthetase, ACP, acyl carrier protein; CoA, coenzyme A; ATP:CL, ATP-citrat lyase; ENR, enoyl-ACP reductase; FAT, fatty acyl-ACP thioesterase; HD, 3-hydroxyacyl-ACP dehydratase; KAR, 3-ketoacyl-ACP reductase; KAS, 3-ketoacyl-ACP synthase; LACS, long chain acyl CoA synthetase; MAT, malonyl-CoA:ACP transacylase; ME, malic enzyme; PDC, pyruvate dehydrogenase complex; PUFA, polyunsaturated fatty acid; TAG, triacylglyceride; TCA, tricarboxylic acid. Different MEs possess different localizations (plastidial, mitochondrial). For simplicity, ME is placed in the cytosol. Modified after [22,65,99].

*Phaedactylum tricornutum* is one of the diatoms best studied at the molecular level. This is due to the sequencing of the 27.45 Mb genome in 2008 [86]. Several studies have been published that have used transcriptomics (microarrays, RNAseq) in combination with metabolomics and related them to physiological/biochemical responses of the photosynthetic system, the lipid metabolism and the carbon flux network [17,87,88,89]. It has been shown that *P. tricornutum* tends to reduce carbohydrate content and increase lipid content under nitrogen starvation compared to non-nutrition limitation, indicating a metabolic switch to lipid accumulation [80,90]. To get more insights into the transcriptional regulation of genes involved in the FA metabolism we performed a co-expression study based on assembled microarray data available on the Gene Expression Omnibus (GEO), NCBI, from the model organism *P. tricornutum*. The unweighted co-expression network (see Figure 3) identified 106 genes that encode enzymes coupled to the FA metabolism and the tricarboxylic acid (TCA) cycle. DiatomCyc [91] and Kyoto Encyclopedia of Genes and Genomes [92] were used for identification of the co-expression cluster, for biochemical pathway analysis, and for protein identification. In the following paragraphs, the genes identified in the *n*-3 LC-PUFAs synthesis and the co-expression cluster (Figure 3) will be discussed. An overview of the primary lipid metabolism in most Chromista, including synthesis of FAs, TAGs and *n*-3 LC-PUFAs, required cofactors, and the end products are presented in Figure 4.

### 5.1. The TCA Cycle and β-Oxidation

To supply the cell with energy and reduced carbon, pyruvate is converted to acetyl-CoA, which enters the TCA cycle to form ATP and carbon skeletons for the anabolic pathways. In *P. tricornutum* the genes encoding enzymes of the TCA cycle show a diurnal regulation pattern when the algae are grown in a day/night cycle [97]. An increased activity of the TCA genes at the beginning of the dark period and co-regulation with genes coupled to cell division suggests that the TCA cycle provide energy for the cell division processes [97]. During the day when cells received their energy via photosynthesis, the TCA cycle associated genes exhibited limited regulation [97]. Figure 4 illustrates that acetyl-CoA is the precursor for both the TCA cycle and the FA synthesis, and that the intermediates of the TCA cycle can be precursors and cofactors in the FA synthesis. Because of this versatile role, the genes of the TCA cycle were included in the cluster analysis. The cluster analysis reveals that the TCA cycle genes had a high degree of co-expression with genes coupled with the FA metabolism. By visual inspection, the cluster can be divided into two subclusters (see Figure 3). Also, genes encoding for mitochondrial enzymes involved in both the FA elongation (C4 < *n* < C16) and the β-oxidation are in this cluster. Whereas oxidation of FA in algae can take place in the mitochondria and the peroxisome, Subcluster 1 represents mostly genes encoding enzymes of the mitochondrial β-oxidation (*ACD1*, *FadJ*, *Phatr2_55192* (enoyl-CoA hydratase), *Phatr2_45947* (3-ketoacyl-CoA thiolase) and *KCT3*). A tight co-regulation between genes coupled to the β-oxidation and the TCA cycle as shown in the cluster is likely, and it has been suggested that oxidation of FAs most likely provides the TCA cycle with acetyl-CoA during dark periods without photosynthetic activity [97]. Malate represents an intermediate of the TCA cycle and can deliver CO_2_ for the plastidial Ribulose-1,5-bisphosphate carboxylase (Rubisco) by the activity of the malic enzymes (ME) [100]. The NAD(P)-dependent ME catalyzes the conversion of malate to pyruvate and provides NAD(P)H for the cell. The irreversible decarboxylation products NAD(P)H and pyruvate can be utilized in the FA synthesis. An overexpression of ME in fungus and yeast species increases the accumulation of lipids [101]. Availability of NADPH can increase the reaction velocity of NADPH-requiring enzymes involved in FA synthesis such as acetyl-CoA carboxylase (ACCase) and ATP citrate lyase (ATP:CL) (Figure 4). FA synthesis is an energy demanding process due to the activity of elongases and desaturases. For instance, the formation of a C18 FA requires 54 NADPH from oxygenic photosynthesis [17]. Biochemical studies with the eustigmatophyceae *N. salina*, grown in batch and continuous cultures, indicated that lipid accumulation is controlled by the availability of NADPH [99]. Transcriptomic studies in N-depleted cells of *P. tricornutum* showed strong up-regulation of the gene producing a predicted plastidial NADP-dependent ME (*Phatr2_51970*) [17]. Such findings indicate that the ME genes are involved in lipid synthesis of *P. tricornutum*. Overexpression of the *Phatr2_51970* may be one avenue to provide electrons which can be channeled into FA synthesis. Mitochondrial malate can also be converted in the mitochondrion or cytosol into pyruvate [99,100]. Therefore, predicted mitochondrial NAD(P)-dependent ME are encoded by *Phatr2_27477* and *ME1* (*Phatr2_54082*), the latter being part of Subcluster 1.

Another intermediate of the TCA cycle serving as a precursor for the *n*-3 LC-PUFA synthesis is citrate. Kinetic profiles and activity studies have revealed that the eustigmatophyceae *N. salina* is able to convert sugar via citrate to lipids [99]. A high activity of ATP-citrate lyase (ATP:CL), encoded by *ACL*, may provide acetyl-CoA for the FA synthesis.

Mixotrophic grown cultures of heterokonts such as *Nannochloropsis* sp., *Dictyopteris membranacea*, *Navicula saprophila* and *P. tricornutum* can incorporate acetate directly into lipids [87,102,103,104]. The acetyl-CoA synthetase (ACS) converts acetate to plastidial acetyl-CoA (Figure 4). In *Nannochloropsis* sp., the acetate uptake is light-dependent. In [^14^C]-acetate labeling studies more than half of the acetate was incorporated into long chain FAs and PC [14,103]. When acetate is abundant in mixotrophic conditions, the lipid levels and subsequently the EPA levels increased by 25% in *N. saprophila* (phototrophic conditions: 27.2 and mixotrophic; 34.6 mg EPA/g biomass) [102].

To conclude, our data shows that the genes of the TCA cycle are co-expressed with the genes of the FA synthesis and may therefore be targeted to improve lipid production.

### 5.2. *De Novo* Fatty Acid Synthesis and Hexadecatrienoic Acid Synthesis

The *de novo* FA synthesis and further processing of one of the most abundant FA in *P. tricornutum*, hexadecatrienoic acid (16:3*n*-4), takes place in the plastid [105]. Thus, acetyl-CoA is the starting point for the FA synthesis in the chloroplast and the ER. Plastidial and cytosolic acetyl-CoA can be provided by the conversion of pyruvate, citrate or acetate. Acetyl-CoA in turn is then converted by the acetyl-CoA carboxylase (ACCase) to malonyl-CoA. In eukaryotes, the ACCase possesses three functional domains; one *N*-terminal biotin carboxylase, one central acetyl-CoA carboxylase and one C-terminal α-carboxyltransferase domain (α-CT). In most plants, the ACCase has an additional chloroplast-encoded subunit, the β-carboxyltransferase (β-CT). To our knowledge, this subunit has not been reported in Chromista which all have homomeric ACCases. The three domains of the homomeric ACCase are located on a multifunctional polypeptide encoded by a nuclear gene [106]. The diatoms *T. pseudonana* and *P. tricornutum* contain two homomeric ACCases, one in the plastid (*ACC1*) and the other in the cytosol (*ACC2*) [106]. The haptophyte *I. galbana*, in turn, has only one plastidial homomeric ACCase. In plants and maybe also in Chromista, the plastidial ACCase (*ACC1*) converts malonyl-CoA for the *de novo* FA synthesis, whereas the cytosolic ACCase (*ACC2*) generates a malonyl-CoA pool as intermediates for the FA elongation [107]. In the co-expression network, both genes are present in Subcluster 2. Although the ACCase is an important enzyme in *de novo* FA synthesis, increased expression of the plastidial ACCase gene *acc1* of the diatoms *Cyclotella cryptica* and *N. saprophila* did not result in increased FA content, indicating inhibition of regulatory processes within the FA synthesis [108,109].

*P. tricornutum* possesses the type II fatty acid synthase (FAS) with discrete, monofunctional enzymes encoded by distinct genes [110]. In the FA biosynthesis pathway, malonylation of malonyl-acyl carrier protein (malonyl-ACP) is carried out by malonyl-CoA:ACP transacylase encoded by *FABD*. Malonyl-ACP is used in the cyclic condensation reactions to extend the acyl group to middle chain length fatty acids (<C18) in the prevalent, plastidial *de novo* FA synthesis [111,112]. The prolonged acyl-ACP is released for further processing by an acyl-ACP thioesterase located in the chloroplast envelope. This thioesterase (TE) has not been characterized in *P. tricornutum*.

**Figure 5:**
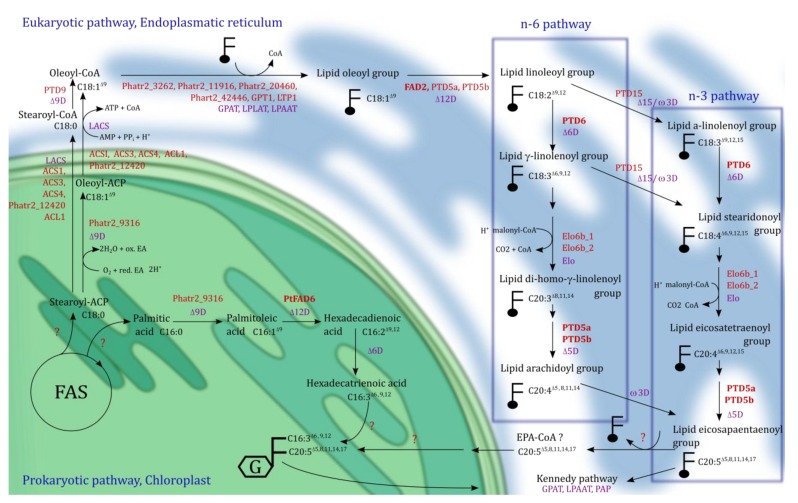
Overview of FA and LC-PUFA synthesis in *P. tricornutum.* Shown are the hypothetical *de novo* fatty acid synthesis (FAS) and the HTA (16:3*n*-4) synthesis plastidial (green) and the EPA (20:5*n*-3) synthesis at the ER membrane (blue) with further incorporation at the *sn-*1 and *sn-*2 position of glycosylglycerides (in plastid or Kennedy pathway in ER). Purple: Long chain acyl-coenzyme A (CoA) synthetases (LACS), lysophospholipid acyltransferases (LPLAT), acyl-CoA:glycerol-3-phosphate acyltransferase (GPAT) and acyl-CoA:lysophosphatidic acyltransferase (LPAAT), phosphatidic acid phosphatase (PAP), elongases (Elo) and desaturases (ΔD or ωD); Red: putative genes, enzymes encoded by genes that have been identified are marked in bold. The other genes are predicted for EPA synthesis from transcriptional data. The co-factors for the desaturases are not indicated. At the ER, the FA are available as acyl-CoA or linked to a glycerol-backbone such as PC indicated by . MGDG is indicated by a glycerol-backbone with a framed G. Before and after elongation, the FA has to be de-linking and re-linking from the glycerol-backbone indicated with two consecutive arrows.Question marks indicate that the reaction and the involved enzymes are not predicted. Modified after [28,113].

In the presence of oxygen, desaturases can saturate FAs into unsaturated FAs. Two types of desaturases can be distinguished; (1) The front-end desaturase contains an *N*-terminal cytochrom b5-domain and inserts the new double bond between the FA carboxyl group and a possible existing double bond. (2) The ω6/ω3 desaturase inserts a new double bond between the FA methyl end and a pre-existing double bond [114]. An example for an identified front-end desaturase in *P. tricornutum* is the high substrate specific plastidial-located Δ12 desaturase, PtFAD6, synthesizes palmitoleic acid (POA, 16:1*n*-7) to hexadecadienoic acid (HAD, 16:2*n*-4), indicated in Figure 5 [105]. Figure 5 shows the synthesis of FA in *P. tricornutum*, especially the putative EPA (20:5*n*-3) and HTA (16:*3n*-4) pathway and genes, which have been either identified or predicted to be involved in these pathways.

Ten out of 12 genes involved in the *de novo* FA and HTA synthesis show a tight co-regulation in Subcluster 2. In the day/night cycle study, these genes as well as *Phatr2_9316* and *Phatr2_50443* showed a strong co-regulation pattern [97]. The maximum expression level of these genes in the beginning of the light period indicates that the FA synthesis uses electrons generated by the photosynthetic machinery. Throughout the day, when energy is needed in other processes, genes coupled to the FA synthesis are downregulated. In the beginning of the light period no neutral lipids were detected [97]. This may support the suggestion that increased availability of NADPH could lead to higher synthesis of FAs.

### 5.3. Acyl-CoA Activator and EPA Synthesis

FA synthesis, membrane glycerolipid synthesis, and β-oxidation require the FA to be present in the acyl-CoA form [115]. The acyl-coenzyme A (CoA) synthetases (ACS) regulate in each compartment the internal acyl-CoA pools by esterification of FAs to CoA. The localization of the pools is maintained due to acyl-CoAs not being able to cross the intracellular membranes [116]. In *P. tricornutum* genes encoding, five long chain acyl-CoA synthetases (LACS) have been predicted (*ACS1*, *ACS3*, *ACS4*, *ACL1* and *Phatr2_12420*). The ATP citrate lyase *ACL1* contains a predicted peroxisomal targeting signal, whereas the other LACS genes exhibit no predicted transmembrane domain, and their localizations are unclear. To our knowledge, the predicted LACSs of *P. tricornutum* have not been functionally characterized.

The cytosolic acyl-CoA pool provides C18 FAs for the extra-chloroplastic EPA synthesis. During the EPA synthesis, the characterized membrane-bound desaturases PTD5, PTD6 and FAD2 appear to be located in the ER membrane and facing the cytosol (Figure 5) [113]. Desaturation of stearic acid (SA, 18:0) to oleic acid (OA, 18:1*n*-9) can take place through a Δ9 desaturase in the chloroplast or the cytosol in *P. tricornutum*. In the plastid, the stearoyl-ACP ∆9 desaturase (*Phatr2_9316*) could convert the reaction, whereas the ER-bound ∆9 desaturase PTD9 may be responsible for the endoplasmatic desaturation by utilizing acyl-CoA as the substrate. Transport of either SA (18:0) or OA (18:1*n*-9) into the cytosol and subsequent activation by the ACSs is necessary. Except the ∆9 desaturase *Phatr2_9316*, ER-located desaturases utilize acyl-lipids (FAs linked to the glycerol-backbone PC) as substrates [28,70,113]. In addition to the chemical character of the glycerol-backbone, the *sn*-position at the molecule also influences desaturation, *i.e.*, the PTD5 and PTD6 had the highest desaturation activity toward the *sn-2* position at PC. However, the ∆12 desaturase FAD2, encoded by *FAD2*, desaturates different glycerol-backbones such as PC and MGDG with similar efficiency [113]. FAD2 possesses high substrate specificity towards OA (18:1*n*-9) (50% conversion) [105]. The acyltransferases residing in the ER are required for the esterification of FAs to the desired *sn*-position of the glycerol-backbone. Most likely the acyltransferases involved in the *n*-3 LC-PUFA synthesis are similar to those in plants (lysophospholipid acyltransferases (LPLAT), acyl-CoA:glycerol-3-phosphate acyltransferase (GPAT) and acyl-CoA:lysophosphatidic acyltransferase (LPAAT)) [117,118]. The latter two represent enzymes of the Kennedy pathway in the ER [119]. One candidate LPLAT (encoded by *Phatr2_20460*) and a LPAAT (encoded by *Phatr2_45551*) have been found in *P. tricornutum*, as well as several GPATs; *GPT1*, *LTP1*, *Phatr2_3262*, *Phatr2_11916*, *Phatr2_42446*. Acyltransferases in microalgae are not well characterized. Therefore, it is uncertain to what extend they control the lipid composition by substrate specificity and relative activity, but a high influence in *n*-3 LC-PUFA synthesis is predicted [118,120].

In *P. tricornutum* EPA is most likely synthesized via a combination of the *n*-6 and *n*-3 pathway [105]. According to Domergue *et al.* (2003), PTD6 converts linoleoyl-PC (18:2*n*-6) to the γ-linolenoyl-PC (GLA, 18:3*n*-6), which then is catalyzed to stearidonoyl-PC (STA, 18:4*n*-3) by a Δ15/ω3 desaturase [121]*.* To be elongated, the FA has to be transferred from the acyl-glycerol-backbone to the acyl-CoA form and afterwards esterified back to the glycerol-backbone by lipase and acyltransferases activities, respectively. Due to the substrate preference of elongases and the need of malonyl-CoA and NADPH, elongases are generally limiting factors in pathway fluxes [25]. The ∆6 elongases in *P. tricornutum* encoded by *ELO6b_1* and *ELO6b_2* are utilizing acyl-CoA as substrate and prolong stearidonoyl-PC (STA, 18:4*n*-3) to eicosatetraenoyl-PC (ETA, 20:4*n*-3). The last desaturation step through the PTD5 desaturase leads to formation of eicosapentaenoyl-PC (EPA, 20:5*n*-3) [121]. Five genes predicted to be involved in the EPA pathway (*FAD2*, *PTD6*, *PTD5b*, *PTD5a*, *ELO6b_2*) have highly co-regulated expression patterns (Figure 3)*.* An alternative *n*-3 pathway involving a ∆9 elongase and ∆8 desaturase was suggested by Arao T. *et al.* (1994) after a ^14^C-FA pulse-chase experiment [122]. In this pathway, ALA (18:3*n*-3) is elongated to eicosatrienoic acid (ETrA, 20:3*n*-3) and further desaturated to ETA (20:4*n*-3), which then is desaturated by PTD5 to EPA (20:5*n*-3). Enzymes involved in this ∆9 elongase and ∆8 desaturase pathway are not identified in *P. tricornutum*, but might be among the elongases and desaturases predicted in Table 1.

**Table 1:** Predicted elongases and desaturases in *P. tricornutum* shown with gene number, enzyme name and predicted annotation*.* Information collected from NCBI, Diatomcyc and KEGG.

*Gene Number Enzyme Name Annotation*			Present in Cluster (Figure 3)	
**Elongases predicted to be membrane bound**				
*Phatr2_16376*	-	Long chain fatty acid elongase		**√**
*Phatr2_9255*	-	Polyunsaturated fatty acid elongase		**√**
*Phatr2_49867*	-	Long chain fatty acid elongase		
*Phatr2_34485*	Elo5b	Long chain fatty acid elongases, membrane bound		**√**
*Phatr2_25360*	-	Probably a short-chain dehydrogenase/reductase		
**Desaturases predicted to be microsomal or membrane bound**				
*Phatr2_55137*	-	Fatty acid desaturase		**√**
*Phatr2_50443*	-	Fatty acid desaturase, cytochrome b5 motif		**√**
*Phatr2_46275*	-	Fatty acid desaturase, putative		
*Phatr2_44622*	-	Fatty acid desaturase		
*Phatr2_22510*	-	Fatty acid desaturase, cytochrome b5 motif		

*P. tricornutum* possesses diverse pathways for EPA synthesis but with few characterized enzymes. The complexity of the pathway originates from the elongation and desaturation steps and the substrate specificities of the associated enzymes which have to switch between the acyl-CoA and acyl-glycerol-backbone pools.

### 5.4. EPA Incorporation into Galactosylglycerides and Triacylglycerides

After elongation and desaturation, EPA is incorporated into the membrane. Although the final location of EPA is to be incorporated into a lipid class, different paths can be perceived as dependent on whether EPA is available as a free FA or linked to a glycerol-backbone. Preferences for the EPA trafficking require further investigation, because it is a complex process. This is especially the case in algae like Chromista where the chloroplasts have four membranes. Free EPA will be linked to PE, PC or DGTS molecules, and then processed through the Kennedy pathway or imported into the plastid and incorporated into glycolipids [123,124]. In the Kennedy pathway, GPAT performs the initial step by adding acyl-CoA to the *sn-*1 position of the glycerol-3-phosphate [119]. The lysophosphatidic acid (LPA) formed is converted by lysophosphatidate acyltransferase (LPAAT) into phosphatidic acid (PA) by adding a second FA to the *sn-*2 position. Diacylglycerol (DAG) is formed by dephosphorylation of the *sn-*3 position through the activity of phosphatidic acid phosphatase (PAP) [119]. PA and DAG as well as other phospholipids can act as precursors for the production of sulfolipids and galactosylglycerides [29]. The incorporation of EPA in sulfolipids is processed by sulfolipid sulfoquinovosyldiacylglycerol synthase (SQD2). MGD and DGD synthases, which also mediate remodeling of membrane lipids in plants, catalyze the final step to synthesize MGDG and DGDG [125]. Several putative genes involved in phospholipid, sulfolipid and galactosylglyceride synthesis as well as in the Kennedy pathway are co-expressed within the cluster *(Phatr2_20460*, *Phatr2_12431*, *Phatr2_33864*, *SQD1*, *Phatr2_33864*, *Phatr2_11390*, *Phatr2_54168*, *Phatr2_14125*). Additionally, three genes encoding glycerol-3-phosphate dehydrogenases are also expressed within the cluster (*Phatr2_12726*, *Phatr2_38067*, *Phatr2_8975*). The glycerol-3-phosphate dehydrogenases produce glycerol-3-phosphate from an intermediate of the glycolysis.

TAGs in *P. tricornutum* are a second EPA pool that is not commonly found in all algae. TAG accumulation mostly occurs under sub-optimal environmental conditions. It is generally assumed that only small amounts of EPA are incorporated into TAGs via synthesis of EPA. Remodeling of FAs from membranes is the main contributor of EPA in TAG [87]. Alternative pathways to convert membrane lipids to TAGs have been shown in both eukaryotes and prokaryotes, and involve phospholipase and phospholipid/diacylglycerol acyltransferase (PDAT). Mus *et al.* (2013) showed that the expression of a PDAT and/or a putative phospholipases increased in *P. tricornutum* under nitrogen starvation, and remodeling of lipid classes such as PC, PE or galactosylipids contributed to lipid accumulation [87]. To regulate EPA production, it is important to understand the mechanisms that determine in what lipid fraction EPA is present. EPA is not present as a free FA but is either incorporated in the membranes or accumulated in TAG.

### 5.5. Genetic Drivers for *n*-3 LC-PUFA Synthesis

It is important to identified genetic drivers for increased *n*-3 LC-PUFA synthesis as they can be used in different optimization approaches such as selective breeding, conditioning and metabolic engineering to enhance the *n*-3 LC-PUFA production in Chromista. Genetic transformation methods based on overexpression and knockdown techniques are already developed for *P. tricornutum* [126,127]. Thus, metabolic engineering approaches may be pursued to increase the amount of EPA (20:5*n*-3). According to Hoffman *et al.* (2008), five main strategies are applicable for engineering of the FA synthesis in both plants and yeast; (1) Increasing the precursor pool, (2) Inhibition of β-oxidation or lipase hydrolysis, (3) Overexpression of FA biosynthetic enzymes, (4) Regulation of thioestarase (TE) for optimizing the FA chain length, and (5) Regulation or introduction of desaturases to control the saturation profile [25].

Following these strategies in *P. tricornutum*, several target genes can be identified partly from the co-expression cluster and from the literature. Strategy 1 has a wide range of targets. Increasing availability of NADPH by overexpression of the ME produced by *Phatr2_51970* might be suitable to provide reduction equivalents for FA synthesis [17]. Overexpressing acetyl-CoA by *ACL* (ATP:CL) and *Phatr2_22974* (acetyl-CoA synthetase) may establish a larger acetyl-CoA pool in the plastid and the cytosol, respectively [22,99]. A larger precursor-pool may also require an increase in transporters, such as the putative plastidal sodium-dependent pyruvate transporter (*Phatr2_3046*) and the acetyl-CoA transporter (*Phatr2_54049*). Both these transporters show co-expression in Subcluster 2 (Figure 3). Also, a possible bottleneck in *n*-3 LC-PUFA synthesis is the qualitative and quantitative acyl-CoA pool. The acyl-CoA pool in turn is dependent on the activity of LACSs regulating the activation of free FAs. In order to provide a suitable acyl-CoA pool the specific activities of LACSs from *P. tricornutum* have to be characterized. Potential targets are the putative *ACS3* and *Phatr2_12420*, which are co-regulated in the Subcluster 2 (Figure 3 and Figure 5). Several ACS in different Chromista have been isolated and characterized already [128]. When a LACS from *N. oculata* was expressed in a LACS-deficient *Saccaromyces cerevisiae* strain, it showed high activity for long chain FA such as eicosadienoic acid (EDA, 20:2*n*-6), LA (18:2*n*-6), SA (18:0) and middle chain unsaturated FA [129]. Reverse transcription-PCR analysis of two LACSs from *Thalassiosira pseudonana* encoded by *TplacsA* and *TplacsI* appeared to be constitutively expressed during cell cultivation [130]. Co-expression of *TplacsI* in a yeast deletion strain (faa4D) TpLACSA showed a broad substrate specificity by inducing the formation of several PUFAs (18:3*n*-6, 18:4*n*-3, 20:4*n*-6, 20:5*n*-3, 22:6*n*-3), but with highest activity towards DHA (22:6*n*-3). The TpLACSA has been shown to be active during the incorporation of DHA in TAGs [130]. Due to their high substrate specificity, LACS are after identification suitable target genes for increasing the synthesis of PUFAs [109]. Overexpression of TpLACSA from *T. pseudonana* might improve the incorporation and production of *n*-3 LC-PUFAs in *P. tricornutum*.

Genes of the β-oxidation pathway are suitable targets for creating knockdown mutants (Strategy 2), but complete elimination of the lipid degradation path may lead to negative effects on the growth and the health of the cell [22,131]. For example, in *Arabidopsis thaliana* the gene-knockouts of both peroxisomal LACS involved in β-oxidation resulted in an alteration of the FA composition, (an increase of monounsaturated long chain FA) and growth inhibition, which was compensated by supply of an external carbon source [131]. Most genes in β-oxidation of *P. tricornutum* are single copy genes. Therefore, knockouts of peroxisomal or mitochondrial localized genes such as *ACD1* and *FAO1* would probably be lethal and strategies of inhibiting β-oxidation might not be suited for *P. tricornutum*. Initial activation of β-oxidation in plants and yeast involves import of FA into the peroxisome by an ATP-binding cassette (ABC) transporter and activation of these FAs by ACSs [131,132]. A similar system of peroxisomal transport and the activation of FAs might also exist in *P. tricornutum* via the peroxisomal located LACS encoded by *ACL1* and an ABC transporter. LACS as well as an ABC transporter (encoded by *Phatr2_15212*) are co-regulated in the cluster (Figure 3).

If other strategies are used, overexpression of genes in type II FAS (Strategy 3) is probably not required in *P. tricornutum* for increasing *n*-3 LC-PUFA synthesis, because if enough energy and precursors are supplied, an upregulation of genes encoding enzymes involved in *de novo* FA synthesis might occur naturally. For instance, in the beginning of the light period, the FA synthesis is upregulated to serve as a sink for excess electrons [97].

In Strategy 4, the feedback inhibitions caused by long chain fatty acyl-ACPs can be suppressed by overexpression of acyl-ACP TE [109,133]. Overexpression of the acyl-ACP TE with specificity for long chain FAs (18:0, 18:1) may improve the *n*-3 LC-PUFA synthesis. A positive effect of TEs on FA compositions was shown by overexpression of acyl-ACP TE specific for shorter chain FA from plants in *P. tricornutum*, resulting in an increase of saturated FAs for TAG assembling [109]. Gong *et al.* (2011) characterized a 4-hydroxybenzoyl-CoA thioesterase PtTE (*Phart2_33198*) from *P. tricornutum*, with an *N*-terminal mitochondrial targeting peptide [134]. Overexpression in *Escherichia coli* showed activity for C18:1-ACP and lower activity for saturated FAs (16:0, 18:0). Overexpression of *Phart2_33198* (PtTE) in *P. tricornutum* increased the total FA content up to 72%. While PA (16:0) and POA (16:1*n*-7) showed a higher content, EPA (20:5*n*-3) showed only a minor increase [134]. So far no plastidial acyl-ACP TE involved in *n*-3 LC-PUFA synthesis has been identified.

Strategy 5 is based on the regulation and overexpression of desaturases to change the FA profile. Desaturases and elongases from *P. tricornutum* that are not functionally characterized or predicted to be involved in a specific pathway are listed in Table 1 and represent potential targets for identification and later on overexpression. Targets for modifying desaturation activity may be *Phatr2_50443* and *Phatr2_55137*. Interestingly, *P. tricornutum* possesses only one ER acyl-CoA Δ9 desaturase and one plastidial acyl-ACP ∆9 desaturase (Figure 5), whereas in the diatom *Fistulifera* sp., six ∆9 desaturases have been functionally characterized [135]. Four desaturases showed acyl-CoA- and two acyl-ACP dependent ∆9 desaturation activity for C16 and/or C18 FAs, indicating that this diatom has more genes for a possible sustainable FA synthesis [135]. Putative FA elongases encoded by *Phatr2_9255* and *Phatr2_16376* indicated by co-expression in the cluster are additional potential targets (Table 1 and Figure 3). In general, the rate-limiting factor of PUFA production in transgenic plants are acyl-CoA dependent elongases, because of the inefficient exchange of acyl molecules back and forth between the desaturation and elongation steps [25]. An advantage would be if the *n*-3 LC-PUFA pathway possesses acyl-CoA-dependent Δ6 and Δ5 desaturases, similar to mammalian desaturases. However, such acyl-CoA-dependent Δ6 and Δ5 desaturases have only been identified in green algae species, and successfully expression resulting in an increase of the *n*-3 LC-PUFA level has only been seen in plants [25,136].

Regulation of the transesterification steps might be an alternative target for overcoming the elongation bottleneck and could be consider as an additional strategy to the strategy of Hoffmann *et al.* (2008) [25]. Overexpression of genes encoding high substrate specific acyltransferases would lead to a higher esterification rate of acyl-donor to a specific *sn*-position of the glycerol-3-phosphate or the PC. The two ER-localized enzymes of the Kennedy pathway (GPAT and LPAAT) and the LPLAT are responsible for the incorporation of activated FAs into membrane lipids, or precursors of membrane lipids [117,118]. Recently, it was shown that an ER-membrane bound TpGPAT from the marine diatom *T. pseudonana* affected FA composition and accumulation in triacylglycerides and phospholipids by overexpression in a GPAT-deficient yeast strain [128]. In the lipid profile, the amount of PA (16:0) in triacylglycerides and phospholipids increased by 18% and 12%, respectively, whereas monounsaturated FA were reduced. *In vitro* enzyme tests showed that TpGPAT has a high preference towards PA (16:0) and a lower towards ARA (20:4*n*-6) as acyl-donor for esterification of the *sn-*1 position of the glycerol-3-phosphate [128]. Two GPATs of the thraustochytrid *T. aureum*, which were isolated and expressed in plants, exhibited high substrate specificity for the esterification of glycerol-3-phosphate with LC-PUFAs (20:3*n*-6, 20:4*n*-3, 20:5*n*-3, 22:6*n*-3) [137]. Whereas GPATs link FAs to glycerol-3-phosphates backbones, radiolabeling studies of flaxseed indicated that LPAAT links C18 FA to the *sn*-2 position of PC [117,118]. Heterologous LPAATs activities of the eustigmatophyceae *N. oculata* were also measured in plants, and they showed high substrate specificity towards C18 FA or C20 FA [137]. This finding shows that diverse acyltransferases such as the LPAAT and GPAT are probably required in EPA synthesis in *P. tricornutum* for the esterification of EPA to the *sn-*2 position of PC [113]. Also, the lysophosphatidylcholine acyltransferase (LPCAT), belonging to the group of LPLATs, may be involved in esterification steps, because LPCAT in plants converts the acyl-group to either the *sn*-1 or the *sn-*2 position of a PC [118]. LPLATs in plants are part of the so-called acyl-editing, which shuffles FAs between the PC-pool and the DAG-pool, by activities of acyltransferases and phospholipases [138]. In plants, acyl-editing mechanisms are very complex and are possible bottlenecks in the FA synthesis [139]. In the co-expression network (Figure 3), several genes are present and identified as enzymes potentially involved in acyl-editing (*Phatr2_32057*, *Phatr2_33864*, see acyltransferase mentioned in Section 5.3) and so in the EPA pathway of *P. tricornutum*.

To conclude, possible genetic driver such as precursors, cofactors, acyl-CoA dependent elongases, LACSs, lipases and acyltransferases have been identified in *P. tricornutum*, and thus show potential as targets for several approaches. Furthermore, the strategies/genetic drivers above can be used to modify *n*-3 LC-PUFA synthesis in commercial approached Chromista, such as *Nannochloropis* or *Isochrysis*.

## 6. EPA Synthesis in Other Chromista

The EPA synthesis in Chromista differs depending on enzyme affinities for glycerol-backbones and desaturation positions. For instance, most eustigmatophyceae are overall identical to *P. tricornutum*, and synthesize EPA (20:5*n*-3) in ER before incorporation into the galactosylglycerides in the plastid. But in small details eustigmatophyceae species also differ to *P. tricornutum* because they preferable synthesize EPA (20:5*n*-3) via the *n*-6 pathway by a ω3 desaturase activity converting ARA (20:4*n*-6) to EPA (20:5*n*-3) [67,103]. In the eustigmatophyceae *Nannochloropsis* sp. and *M. subterraneus*, it is predicted that this conversion step involves Δ17 desaturase activity, but this is not yet confirmed [67,103]. Eustigmatophyceae are important algae in industrial production today, and the genus *Nannochloropsis* (including six different species) is most promising because of their growth rate, their robustness and their haploid genome which is easy to engineer [140,141,142,143,144]. The major FAs in *Nannochloropsis* species are POA (16:1*n*-7) followed by EPA (20:5*n*-3) and PA (16:0) [77,99,145]. So far, two Δ12 desaturases, two Δ9 desaturases, one ω3 desaturase and several elongases have been shown to be involved in the synthesis of the main FAs in *Nannochloropsis* [146,147]. The characteristics of the desaturases and the elongases were shown by activity studies and overexpression of target genes under regulation of different promoters, which were inserted into the genome via homologous recombination [146]. Two Δ9 desaturases showed to be involved in the synthesis of one of the main FAs of *Nannochloropsis*, POA (16:1*n*-7), by detection of high substrate activity towards PA-CoA (16:0) and PA (16:0) linked to a glycerol-backbone [146]. Contrary to Δ9 desaturases, the two Δ12 desaturases showed different activities. One desaturase is indicated to be involved in the plastidial unsaturation of C16 FAs, and the other Δ12 desaturase likely has OA (18:1*n*-9) as substrate and is probably involved in the EPA synthesis. The ω3 desaturase involved in EPA synthesis showed high substrate preferences towards ARA (20:4*n*-6) and LA (18:2*n*-6) [146]. Overexpression of the ω3 desaturase in *Nannochloropsis* resulted in a large increase of the EPA/ARA ratio, and a small increase of ALA (18:3*n*-3) [146]. *In vivo* ^14^C-C18:1-CoA radiolabeling studies showed C18 FAs desaturation by Δ6 and Δ5 desaturases when the FA was attached to the *sn-*2 position of PC, which is similar to in *P. tricornutum*. In contrast to *P. tricornutum*, positional analysis of labeled lipids indicated acyltransferase activities linking ^14^C-C20:4-CoA to the *sn-*1 and *sn-*2 position of PE [103,148]. Concomitantly, other lipids such as the betaine lipid DGTS and TAG were labeled to a small degree. Also, the acyl moieties C16/C18 PC, C20/C20 PE and C20/C16, C20/C14 DGTS were produced. Consequently, the apparent source of MGDGs is the ER-located acyl-PEs by being converted into DAG and imported into the plastid. Molecular species of DGDGs use DGTS as precursor which has EPA attached at the *sn-*1 position [67].

Whereas most algae utilize PC to assemble glycolipids and synthesize *n*-3 LC-PUFAs, it has been shown that *Nannochloropsis* sp. and *M. subterraneus* utilize PC, PE and DGTS. Conversely, the brown algae *D. membranacea* has complete absence of PC, and the betaine lipid diacylglyceroltrimethylalanine (DGTA) seems to have the role of the PC and therefore plays a key role in the EPA and galactosylglyceride synthesis in *D. membranacea* [104]. DGTA is somewhat neglected when it comes to lipid metabolism, but it seems to be more important for the PUFA synthesis of some species to have a higher abundance of betaine lipids than previously thought [104,149].

Recently, eustigmatophyceae are of more interest which is reflected in the research making progress by identifying genes that encode enzymes involved in the EPA synthesis. Still, no transcriptome data are available and so the lack of characterized genes hampers the manipulation of the Section 5.5 identified genetic drivers.

## 7. DHA Synthesis in Other Chromista

Besides EPA (20:5*n*-3), DHA (20:6*n*-3) is the other high-valuable *n*-3 LC-PUFA which can be found in several Chromista species. These species possess specific Δ5 elongase and Δ4 desaturase activities for the consecutive steps of converting EPA (20:5*n*-3) into DHA (22:6*n*-3). Unlike mammals and fish, Chromista do not produce DHA via the Sprecher pathway by shortening tetracosahexaenoic acid (THA, 24:6*n*-3) in the β-oxidation pathway [150]. Chromista possess a simpler pathway to synthesize DHA (22:6*n*-3). This makes Chromista favorable DHA producers and their enzymes more interesting for metabolic engineering. The main DHA producers among Chromista are the three genera of thraustochytrids (*Thraustochytrium* sp. and *Schizochytrium*, *Aurantiochytrium*) that accumulate high DHA amounts in TAGs under nitrogen depletion [13]. Interestingly, the heterotrophic thraustochytrid *Schizochytrium* sp. (genera renamed to *Aurantiochytrium*) possesses an alternative anaerobic polyketide synthase pathway (PKS) for the *n*-3 LC-PUFA synthesis [151]. A large multifunctional enzyme complex carries out the multitude of individual reactions, utilizing malonyl-CoA and producing free *n*-3 LC-PUFAs [151]. The major free FAs DPA (22:5*n*-6) and DHA (22:6*n*-3) are then activated to acyl-CoA and incorporated into TAGs [152]. To convert the major saturated components of TAGs (14:0 and 16:0), *Aurantiochytrium* additionally contains additionally a short-chain FAS complex [153]. Different from the type II fatty acid synthase (FAS) of *P. tricornutum*, the FAS of *Aurantiochytrium* is a type I FAS synthesized from one or two polypeptides [110]. The synthesized free FAs and the absence of genes homologous to a type II TE may indicate integration of TE activity into the synthase [152]. Because PKS does not require aerobic desaturation, the pathway is energetically favorable compared to the membrane-bond desaturases and elongases [27]. However, it was shown that ATP:CL and ME play key roles in the lipid assembling in *Schizochytrium* sp. S31, by providing acetyl-CoA and NADPH to the FAS and the PKS [154]. The energetically favorable pathway for DHA/*n*-3 LC-PUFA production is not the only pathway in thraustochytrids. *T. aureum* for instance is capable of synthesizing *n*-3 LC-PUFAs through the standard *n*-3 LC-PUFA pathway. ∆5 and ∆12 desaturase activity was detected in the PUFA pathway, indicating that either acyl-CoA substrates are used or acyl-edition occurs after desaturation [155]. A gene-knockdown mutant deficient in ∆12 desaturase showed a change in FA composition, but maintained high production of DHA via the PKS pathway [155]. This indicates that DHA (22:6*n*-3) is synthesized in the PKS pathway, whereas GLA (18:3*n*-6) and EPA (20:5*n*-3) are synthesized in the common PUFA pathway. Further studies on *Thraustochytrium* sp. identified two genes for elongases (*tselo1* and *tselo2*) and a Δ4 desaturase encoding gene to be enzyme is involved in LC-PUFA/DHA synthesis [156,157]. PCR-based gene identification studies revealed that many species within *Thraustochytrium* sp. and *Schizochytrium*, but not *Aurantiochytrium*, possess ∆4 desaturates and Δ5 elongases [158].

Several desaturases in Chromista are characterized, such as the Δ4 desaturase of the haptophyta *I. galbana* or *P. lutheri* [159]. The DHA-producing haptophyta *P. lutheri* contain STA (18:4*n*-3), EPA (20:5*n*-3) and DHA (22:6*n*-3) as the main FAs. *P. lutheri* contain no PC, but have instead betaine lipids (DGCC, DGAT, see Figure 2) [69]. DGCC is the glycerol-backbone for both the transfer of FAs in the plastid and the synthesis of MGDG [69]. Radiolabeling studies indicated that the main common LC-PUFA pathway started at PA (16:0), and involves possibly the *n*-6 pathway with the intermediates DGLA (20:3*n*-6) and ARA (20:4*n*-6) [160]. Through labeling of ^14^C-20:3*n*-6, most radioactivity was detected in EPA (64%–67%) and DHA (9%–21%), which indicate a desaturase that converted either ETA (20:4*n*-3) or ARA (20:4*n*-6) to EPA (20:5*n*-3) [160]. Based on studies in the red algae *P. cruentum* and the freshwater eustigmatophyceae *M. subterraneus*, Δ17 desaturase activity has been proposed in *P. lutheri* [73]. No clear evidence of a Δ17 desaturase in *M. subterraneus* exists; however, such an activity exists in the chloroplast of the red algae *P. cruentum* [73,160]. After lipid-linked ARA (20:4*n*-6) is transported into the chloroplast, it is further desaturated to EPA (20:5*n*-3) and incorporated into galactosylglycerides [73]. The identified Δ5 elongase and Δ4 desaturase encoded by *pavELO* and *Pldes1* can prolong EPA (20:5*n*-3) to DHA (22:6*n*-3) in *P. lutheri* [161]. The gene *pavELO* possesses a unique substrate specificity towards C20 FA (EPA (20:5*n*-3) and ARA (20:4*n*-6)) expressed in yeast. Also, the Δ4 desaturase (encoded by *Pldes1*) showed desaturation activity for generating adrenic acid (ADA, 22:4*n*-6) and DPA (22:5*n*-3) [162].

When it comes to industrial DHA-production strains of Chromista, two different pathways—the PKS and the common PUFA synthesis—are suggested to synthesis DHA (22:6*n*-3). In the genus thraustochytrids similarities to desaturases and elongases in other Chromista were identified, but it is still unknown which pathway thraustochytrids utilize for DHA synthesis.

## 8. Conclusions

Species within Chromista are promising candidates for sustainable production of high-value PUFAs, with different species providing different opportunities and challenges. As the quest for PUFA is increasing, comparative studies will provide exciting advances in our basic knowledge of the metabolism of this group of organisms. This review reflects that the knowledge on biochemistry of lipids and genetic regulation of the FA and lipid synthesis is evolving rapidly, and will continue to do so in the coming years.

Biosynthesis of PUFAs in Chromista has mainly been studied by biochemical/metabolic studies including FA composition analysis, desaturation/elongation activity measurements, pulse-chase radiolabeling and inhibitor studies [28]. In this review, the diatom *P. tricornutum* has been used to exemplify the complexity of *n*-3 LC-PUFA synthesis in Chromista. Full genome sequences, biochemical analysis, and the fact that recent studies demonstrate that homologous recombination is feasible in the challenging diploid genome of *P. tricornutum* makes this diatom a good model organism [86,163]. However, industrial application of *P. tricornutum* is unlikely [22]. Possible bottlenecks in *P. tricornutum* synthesis of *n*-3 LC-PUFAs were identified with the help of a co-expression network study of genes coding for proteins involved in lipid synthesis. Our analysis points to enzymes producing precursors and cofactors (acetyl-CoA pool and NADPH) for the *n*-3 LC-PUFAs synthesis as suitable targets for further improvements in *n*-3 LC-PUFA content by using different approaches such as selective breeding, environmental conditioning and genetic engineering. Other key players in *n*-3 LC-PUFAs synthesis are TE, acyl-CoA-dependent elongases, LACSs and acyltransferases. This knowledge was compared with knowledge from Chromista species with greater interest for industrial applications. In general, genetic drivers could be identified but most genes encoding the genetic drivers are still uncharacterized in *P. tricornutum* and in most Chromista. This hampers the application of, for example, genetic engineering. However, with 15 sequenced available genomes of algae, a new era has started that will result in a rapid improvement of our understanding of transcriptional lipid metabolism and gives us the opportunity to characterize the identified genetic drivers [91,106]. Genetic transformation has been shown to be successful in more than 30 strains of algae, and expression stability can be controlled by endogenous promoters, species-specific codon usage and intron sequences [22]. Currently, most commercial interest for engineering lipid metabolism is on the *Nannochloropsis* genus, which under nitrogen starvation can accumulate more than 55% of cell dry weight as FA with >5% in the form of EPA [145]. The established homologous recombination technique for the haploid genome is a powerful tool to drive lipid research in this organism and also to introduce genes for DHA production in this heterokont [140,141,142,143,144]. Furthermore, it has been shown in *Nannochloropsis* and *Pavlova* that genetic breeding can yield to a higher lipid or *n*-3 LC-PUFA content, respectively [164,165,166]. With the knowledge of the genetic drivers and the biochemical pathway, selective breeding can be improved by easier selection of triggers for creating a genetic bottleneck that selects for cells with a high capacity for lipid production.

It seems clear that manipulation of, for instance, elongases and acyltransferase can lead to a higher *n*-3 LC-PUFA content in biologically interesting Chromista, as it has been shown in the patents of the species of *Nannochloropsis* [146,147]. However, it remains to be seen at which point the algal physiology may be limiting for that approach. Therefore, a combination of approaches such as metabolic engineering, conditioning and selection may be more suitable for both increasing the biomass density and increasing the *n*-3 LC-PUFA content in the biomass.

The intake of *n*-3 LC-PUFAs is a dietary requirement for humans for healthy cognitive development in infants, as well as reducing the risk of chronic diseases for adults. As the need for a sustainable *n*-3 LC-PUFA source increases proportionally to the increase in global populations, more research is required in alternative biological sources, therefore providing a potential long-term source of *n*-3 PUFAs. Even though much research has to be done on the physiological and molecular levels, Chromista are a promising long-term alternative source of *n*-3 LC-PUFAs for humans and fish feed.

## Fatty Acid Nomenclature and Abbreviations

ADA: adrenic acid (22:4*n*-6)
ALA: α-linolenic acid (18:3*n*-3)
ARA: arachidonic acid (20:4*n*-6)
DGLA: dihomo-γ-linolenic acid (20:3*n*-6)
DHA: docosahexaenoic acid (20:6*n*-3)
DPA: docosapentaenoic acid (22:5*n*-3)
EDA: eicosadienoic acid (20:2*n*-6)
EPA: eicosapentaenoic acid (20:5*n*-3)
ETA: eicosatetraenoic acid (20:4*n*-3)
ETrA: eicosatrienoic acid (20:3*n*-3)
FA(s): fatty acid(s)
GLA: γ-linolenic acid (18:3*n*-6)
HAD: hexadecadienoic acid (16:2*n*-4)
HTA: hexadecatrienoic acid (16:3*n*-4)
LA: linoleic acid (18:2*n*-6)
LC-PUFA(s): long chain polyunsaturated fatty acid(s), >C20
MA: myristic acid (14:0)
OA: oleic acid (18:1*n*-9)
PA: Palmitic acid (16:0)
POA: Palmitoleic acid (16:1*n*-7)
PONA: palmitolenic acid (16:2*n*-7)
PUFA(s): polyunsaturated fatty acid(s), >C18
SA: stearic acid (18:0)
STA: stearidonic acid (18:4*n*-3)
THA: tetracosahexaenoic acid (24:6*n*-3)

## Appendix: Definitions and Abbreviations

**Acetyl-CoA carboxylase (ACCase):** Catalyzation of acetyl-CoA to malonyl-CoA in the chloroplast and the cytosol.

**Acyl-CoA synthetase (ACS):** Substrate specific esterification of a FA to a glycerol-backone (see Kennedy pathway).

**Acyl-editing:** Shuffling the FAs between the PC-pool and the DAG-pool by activities of acyltransferases; phospholipid/diacylglycerol acyltransferase (PDAT), lysophospholipid acyltransferases (LPLAT) and phospholipases.

**ATP-citrate lyase (ATP:CL):** Synthesis of citrate to acetyl-CoA.

**Betaine-type glycerolipids:** Membrane compartments; diacylglyceryltrimethylhomoserine (DGTS), diacylglyceryl glucuronide (DGGA), diacylglycerylhydroxymethyltrimethylalanine (DGTA), diacylglycerylcarboxyhydroxymethylcholine (DGCC).

**Galactosylglycerides:** Membrane compartments; eukaryotic and prokaryotic species of Monogalactosyldiacylglycerol (1,2-diacyl-3-*O*-(β-d-galactopyranosyl)-*sn* glycerol, MGDG), digalactosyldiacylglycerol (1,2-diacyl-3-*O*-(α-d-galactopyranosyl)-(1→6)-*O*-β galactopyranosyl *sn*-glycerol, DGDG).

**Glycosylglycerides:** Represent in the thylakoid and the non-photosynthetic membrane; MGDG, DGDG and sulphoquinovosyldiacylglycerol (1,2-diacyl-3-*O*-(6-deoxy-6-sulfo-a-d-glucopyranosyl)-*sn* glycerol, SQDG).

**Fatty acid synthase (FAS):** Synthesize malonyl-ACP to short chain FAs in several condensation steps, type I FAS: FAS synthesized from one or two polypeptides, type II FAS: discrete, monofunctional enzymes encoded by distinct genes.

**Fatty acyl-ACP thioesterase (FAT):** Releases free FAs from acyl-ACPs, synthesized from de novo fatty acid biosynthesis.

**Kennedy pathway:** Esterification of glycerol-3-phosphate by acyl-CoA:glycerol-3-phosphate acyltransferase (GPAT). The formed lysophosphatidic acid (LPA) is converted by acyl-CoA:lysophosphatidic acyltransferase (LPAAT) into phosphatidic acid (PA) by adding a second FA to the *sn-*2 position. Dephosphorylation of the *sn-*3 position forms through the phosphatidic acid phosphatase (PAP) activity. Diacylglycerol (DAG) is formed which can be synthesis to TAG.

**Long chain acyl CoA synthetase (LACS):** Activation of FA by esterification to coenzyme A.

**Malic enzyme (ME):** Possess different localizations (plastidial, mitochondrial), synthesize malate and NAD(P) to pyruvate and NAD(P)H.

**Phosphoglycerides:** Membrane compartments; phosphatidylcholine (PC), phosphatidylglycerol (PG), phosphatidylethanolamine (PE).

**Polyketide synthase (PKS):** Multi-domain enzymes or enzyme complexes that synthesize PUFAs while being attached to ACP-domains.

**Pyruvate dehydrogenase complex (PDC):** Complex of three enzymes transforming pyruvate into acetyl-CoA by a process called pyruvate decarboxylation.

**Thioesterase (TE):** Hydrolyze thioester bonds.

**Triacylglycerides (TAGs)**: Lipid storage compartments in algae, three FAs are esterificated to a glycerol.
